# Instruments to Assess Physical Activity in Primary Education Students with Autism Spectrum Disorder: A Systematic Review

**DOI:** 10.3390/ijerph18094913

**Published:** 2021-05-05

**Authors:** Paula López-Valverde, Javier Rico-Díaz, Martín Barcala-Furelos, Mariacarla Martí-González, Juan L. Martín, Sergio López-García

**Affiliations:** 1 Faculty of Education Sciences, Universidade de Santiago de Compostela, 15782 Santiago de Compostela, Spain; paula262lv@gmail.com; 2 Faculty of Health Sciences, European University of the Atlantic, 39011 Santander, Spain; martin.barcala@uneatlantico.es (M.B.-F.); mariacarla.marti@uneatlantico.es (M.M.-G.); juan.martin@uneatlantico.es (J.L.M.); 3 Faculty of Education, Pontifical University of Salamanca, 37007 Salamanca, Spain; slopezga@upsa.es

**Keywords:** autism spectrum disorder, ASD, physical activity, primary school, children, measurement

## Abstract

The scientific evidence supports that physical inactivity in childhood is a reality throughout the world which generates important consequences in the global development of children. Young people with Autism Spectrum Disorder (ASD), due to the characteristics of the disorder they suffer, constitute a group at risk. Therefore, assessing the levels of physical activity (PA) in this group is fundamental for subsequent decision making and implementation of PA promotion programmes. Consequently, the aim of this systematic review was to identify, summarise and analyse the main instruments used to assess the levels of PA (in terms of time and/or intensity) in primary school children diagnosed with ASD. Scientific articles in English and Spanish published in five databases were reviewed: PsycINFO, WOS, SPORTDiscus, Scopus and PubMed, following the guidelines of the PRISMA statement. Out of the 605 articles identified, 12 met the previously established inclusion criteria. The instruments used by the studies analysed were divided into two main groups: accelerometers and questionnaires. Both showed different strengths and limitations but agreed on the low levels registered of PA in children with ASD. For this reason, it is considered necessary that further research be carried out in this field, as well as the development and implementation of sports programmes adjusted and adapted to the needs and characteristics of the ASD group.

## 1. Introduction

Physical inactivity is a major risk factor for mortality worldwide and there is a high prevalence at all age levels [1]. Its incidence has increased progressively over the last few years, and it is a determining factor in the emergence of non-communicable diseases, such as cardiovascular diseases, cancer or diabetes [2] and respiratory diseases [3]. Furthermore, it contributes to the increase of obesity [3,4], another major current global problem, which has almost tripled its prevalence in recent decades [5].

Children are one of the main groups at risk, as approximately 80% of young people do not meet the recommendations of 60 min of daily moderate-to-high intensity physical activity (PA) [2]. Within this group, young people with Autism Spectrum Disorder (ASD) present an even greater risk of inactivity than their peers with normotypical development, since their particular characteristics, which define the disorder they suffer, limit their own practice of PA [6].

ASD is characterised by difficulties in communication and social interaction and the presence of restricted, repetitive and stereotyped behaviours and interests; motor difficulties and comorbid intellectual disability (ID) are also common [7]. A recent report published by the Centers for Disease Control and Prevention (CDC) estimated that this disorder affects 1 in 59 children [8]. These socio-communicative, behavioural and motor impairments [9,10], combined with stereotypical attitudes, may reduce opportunities to participate in physical activity and sport. This contributes to increased sedentariness among young people with ASD [6] and to the emergence of diseases affecting health [9], such as overweight [11]. More specifically, the significant barriers with regard to social and communication skills can restrict activities where collaboration is essential, such as participation in team sports or group games [12]. This situation, combined with the usual deficits in motor development, leads children with ASD to opt for simple, solitary activities [12]; in addition to contributing to a sedentary lifestyle, this has a negative impact on their already limited social skills [10]. Their problems to adapt themselves to the alterations in routine also affect their level of PA practice, as it usually involves complex and novel stimuli [9].

Despite the difficulties of young people with ASD to participate in physical sports activities, PA practice has proved to be tremendously beneficial for this group: it favours the development of their social skills [13,14], especially in the areas of interaction, cooperation and adaptation [14]; it decreases the stereotypical behaviours associated with the disorder [15]; it reduces motor deficits, demonstrating general improvements in basic motor skills [16]; and it increases the performance of executive function, which is generally impaired in students with ASD, notably improvements in working memory and meta-cognition [17].

As a result, the promotion of PA for children with ASD is imperative from an educational point of view, introducing all the necessary measures and adaptations. Thus, it would be intrinsically essential to assess the level of PA practice (in terms of time and intensity of practice) of students in order to identify existing problems and design the necessary intervention strategies within the educational context [18]. Additionally, in order to assess the activity patterns of young people with ASD, it would be advisable to first select an instrument that, adapted to their specific characteristics, correctly records their PA levels. In this way, the aim of this systematic review was to identify, summarise and analyse the main instruments used to assess the levels of PA (in terms of time and/or intensity) in primary school children diagnosed with ASD.

## 2. Materials and Methods

### 2.1. Search Strategy

This systematic review was developed along the lines of the PRISMA statement [19] and five international databases (PsycINFO, WOS—Web Of Science, SPORTDiscus with Full Text, Scopus and PubMed) were used to conduct the literature search dated 16 May 2020. We started with the search on PsycINFO. The other searches followed (Table 1).

The search strategies included four keyword categories (ASD, PA, measurement and population) and two eligibility criteria (language and document type). The different words configuring the theme categories were combined through the Boolean connector OR. Finally, the different categories were mixed through the AND connector. Table 1 shows the complete search strategy carried out in PsycINFO, which was subsequently used in the other databases, according to the specific characteristics of each one.

### 2.2. Inclusion Criteria

Once the search in the five databases was completed, the final selection of studies was conducted, according to the previously established inclusion criteria. These were: (1) interventions in the school population (6–12 years old); (2) assessment of the level of PA practice (in terms of time and/or intensity) and (3) the use of objective and/or subjective measurement instruments described in detail. Those articles of which the main objective was not the assessment of PA levels in school populations with ASD, but in which this assessment had to be performed (according to the inclusion criteria already described) in order to achieve the main goal of this study, were also included. In addition, a filter was established by language, selecting only those publications in Spanish or English. No filter was used with respect to the date of publication or completion of the study. Comments, correspondence, opinion articles, abstracts and letters to the editor were also excluded.

## 3. Results

### 3.1. Summary of the Studies Included

**Figure 1:**
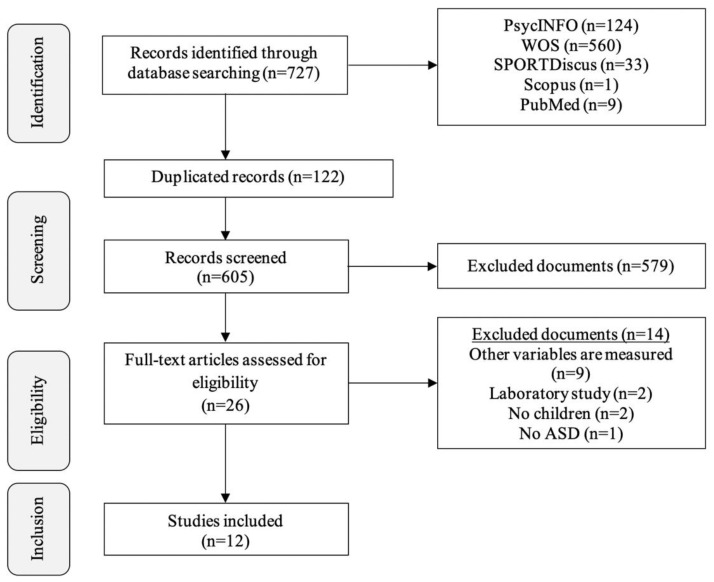
Article selection flowchart.

The initial search identified 727 articles. Out of these, 122 were duplicates; thus, the titles and abstracts of 605 papers were analysed using the inclusion/exclusion criteria. This left 26 articles to be fully read, concluding the inclusion process with 12 articles (Figure 1).

Table 2, Table 3 and Table 4 show, in chronological order, a summary of the most outstanding aspects of the 12 studies which met the inclusion criteria. All of them are journal articles published between 2008 and 2019 assessing the level of PA (time and/or intensity) in children with ASD through objective and/or subjective measurement instruments. In total, 50% of the papers were conducted in the United States.

The total sample analysed included 606 participants. Among them, 421 were schoolchildren with ASD: 316 were boys and 105 were girls. From the remaining 185 participants, 169 were children with normotypical development, 10 showed ASD and 6 had visual impairment (VI). Young people with normotypical development made up the control groups in five of the studies [20,23,25,27,28]; the remaining papers (seven) did not include a control group. As for the diagnosis of ASD, reported in Table 2, six studies did not specify the degree of severity of the disorder [22,23,25,26,28,30], one included only participants with associated ID [29] and five limited the sample to children with high-functioning autism (IQ ≥ 70) [20,21,24,27,31]. Females accounted for 25% of the total sample, while the age of schoolchildren ranged from 3 to 20 years old.

The results of most studies concluded that children with ASD did not meet the recommendations of daily PA and that, when compared to young people with normotypical development, they were less physically active. On top of that, a high percentage of studies held that girls participated in less PA than boys and that the older the child, the more likely they were to be inactive (Table 4). These data were collected through different PA measurement instruments (Table 3). Specifically, the instruments used in the different studies of the review were grouped into two categories; seven articles made use of objective assessment instruments [20,21,23,26,29,31], four used subjective methods [22,24,25,28] and one combined both [27].

### 3.2. Objective Measurement Instruments

The accelerometer, as shown in Table 3, was the common factor in all objective measurements. Only Pan’s study [20] made use of the uniaxial accelerometer, while all others used triaxial devices. The location of placement depended on the characteristics of each accelerometer. In five studies the measurements by accelerometry were of 7 days and in the remaining three they were lower than this figure: more specifically, four [26], five [20] and six [29]. Participants wore accelerometers 24 h a day in four studies, during waking state hours in two [23,26] and only during the school day in two others [20,29].

In three studies, the use of the accelerometer was accompanied by a logbook, which families and/or teachers had to fill out throughout the intervention in order to record the times at which children did not use the device [21,23,27]. Two other articles also used complementary instruments: a questionnaire to measure sedentary behaviour [21] and a questionnaire to measure time of exposure to screens [31].

### 3.3. Subjective Measurement Instruments

Subjective assessment methods were applied by a total of five studies, always using the questionnaire (Table 3). Only families were asked to complete the questionnaire in two studies [22,25], in two other studies the questionnaire was completed by both families and children with ASD [27,28], and one study combined the participation of families and teachers [24].

All articles included issues related to the frequency and intensity of children’s PA, using the MEND [22], PAQ-C [27], CHAMP [25] and GLTEQ adaptations [24,28]. For complementary instruments, two studies used a questionnaire to measure screen time [25,28] and one incorporated a daily activity log to record children’s type of play (individual or social) [24]. In addition, two articles used a checklist to record barriers in the practice of PA [24,25].

## 4. Discussion

The regular practice of PA plays a key role in the proper growth and development of all children, both physically and mentally. Its many benefits are especially significant in the ASD group, showing significant improvements in their difficulties. Therefore, by being aware of the influence of PA on children with ASD, its measurement (in terms of time and/or intensity) constitutes the first step to achieve a more active lifestyle, since only by identifying existing problems can measures and strategies be designed to promote greater PA practice. In this regard, this systematic review has aimed to identify and analyse the instruments used to assess the levels of PA (in terms of time and/or intensity) in Primary Education schoolchildren diagnosed with ASD.

The instruments used by the articles included in the review for assessing the PA among young people with ASD were divided into two main categories: objective and subjective. In the first one, used by a total of eight studies, the accelerometer stood out; while in the second group of studies (five), the questionnaire was the most demanded instrument.

### 4.1. Accelerometer

The accelerometer directly measures PA, its duration and intensity, and even the time children spent in sedentary behaviour. Moreover, they can be used in all activities and during sleep hours, water activities being an exception (in many cases).

Several models of accelerometers can be distinguished. In fact, in the eight articles of the review using the accelerometer, six different archetypes were found (GT1M, GT3X, GTX3+, Pro-3 SenseWear, Omron/HJA-750C and GT9X), among which one uniaxial and five triaxial accelerometers were identified. Despite the differences, the results of the instruments were similar, as they all recorded a low level of PA in participants with ASD. This finding is consistent with the study by Vanhelst et al. [32], who, when comparing the feasibility of a uniaxial (WG1M) and a triaxial (RT3) accelerometer to assess PA in a group of 13–16-year-old adolescents, concluded that the two types of accelerometers did not differ in the measurement of PA. However, other publications disagree, arguing that triaxial accelerometers provide more information and are more accurate when assessing PA and the energy expenditure of young people [33]. The placement of the device is a variable to be considered. Most of the studies analysed installed the device on the hips of participants, which coincides with most scientific research, as the hip has traditionally been the most common site of placement [34]. However, recent studies concluded that subject acceptance, especially for children, is higher when the accelerometer is placed on the wrist or ankle [35]. In fact, this is precisely where one of the main limitations of the accelerometer in young people with ASD lies: the ability of the subjects to withstand the device [36]. Nevertheless, according to the results described by the papers included in this systematic review, only one research student was excluded because he did not use the device long enough [31]. The remaining studies did not describe any difficulties with the use of the instrument by children with ASD. The good acceptance of the accelerometer by these children may have been influenced by some of the conditions of the studies, such as the low presence of participants with associated ID. Thus, it is considered difficult to generalise the usefulness of the instrument for the entire disorder, especially taking into consideration the findings of other interventions such as that of Oreskovic et al. [37], who found that the most common reason for abandonment in their study, among the 9 participants out of the initial 21, was the inability to tolerate the device due to the typical sensory problems of ASD.

These drawbacks are also compounded by the stereotypical (non-functional) movements associated with children with ASD, as the accelerometer may make it difficult to differentiate between voluntary and non-voluntary behaviours [21,31]. Furthermore, other publications have also pointed to the high cost of accelerometers as one of the main reasons for their occasional rejection [36], in favour of other cheaper and objective instruments (pedometers) or subjective tests such as self-report questionnaires [38].

As for the pedometer, no information is recorded about the frequency, duration and intensity of activity, so its use to assess young people’s free participation in PA is limited [33]. PA bracelets also stand out as being easy to use, widely accepted by children and cheaper than accelerometers [39].

### 4.2. Questionnaire

The questionnaires used in the studies reviewed were mainly completed by families; while this does not eliminate the bias inherent to the subjectivity of these qualitative methods, it does mean that responses are not influenced by the usual planning and communication difficulties of children with ASD [7]. Indeed, other research argues that self-report questionnaires should not be used in childhood, especially before the age of 10, and that families and teachers should be responsible for carrying them out [38]. In terms of teacher participation, only one of the studies included in this review involved teachers [24], which is essential to implement PA in schools.

According to another study [40], PA questionnaires should ask participants for information about the PA carried out during the week and during the weekend, in order to be able to assess the total amount of PA. In the study by Bricout et al. [27], the PAQ-C was used, including questions aimed at identifying PA performed during weekdays and weekends. This questionnaire showed a moderate-high reliability with respect to PA quantified through accelerometry, good internal consistency and reliability over 4 months of intervention [41]. However, the GLTEQ questionnaire used by two of the studies included in the review [24,28] was called into question by the analysis of Cancela-Carral et al. [40], whose results showed a low degree of correlation with objective measures. There are many other questionnaires which, although not validated in children with ASD, are suitable for the assessment of PA in childhood and adolescence [39]. Therefore, due to the characteristics of children with ASD, their reliability should be assessed in this population.

The subjective nature of the questionnaire, as opposed to the objective nature of the accelerometer, is the main drawback of this evaluation method since, as all the interventions analysed show, the difficulty to accurately remember the different activities carried out in a certain period of time is a major limitation when it comes to notifying the safety of the instrument. Other studies which have investigated the reliability and validity of PA questionnaires in children have highlighted this limitation [38,42], ranking it as the greatest barrier when using the questionnaire. However, this method of measurement has notable advantages which render it the most commonly used resource [40], highlighting its low economic cost [38], ease of administration, capacity for simultaneous evaluation in large samples [40] and the possibility of learning about the context and type of activity [42].

### 4.3. Physical Activity Levels

Regarding PA levels, all instruments, objective and subjective, detected a low level of PA in children with ASD; they did not comply with the recommendations of 60 min of daily moderate-to-high intensity PA and were physically less active than their normotypically developing peers. Some studies included in the review argued that girls participated in less PA than boys [21,24,28] and that as the age of the participants increased, PA practice decreased [20,21,24]. Additionally, it should be noted that the study by Memari et al. [24] reported a clear preference of participants with ASD for solitary play, which is supported by the results of Healy et al. [28], who reported that children with the disorder participate in fewer sports than their peers without ASD. Must et al. [25], on the other hand, detected more barriers towards the PA practice in students with ASD than in schoolchildren with normotypical development.

### 4.4. Study Limitations

This systematic review was subjected to a number of limitations. Firstly, it should be mentioned that the studies were only searched in the databases mentioned, and relevant information may have remained unanalysed. In addition, a filter was established by language, selecting only those publications in Spanish or English, and therefore, documents written in other languages, although they may be of interest, were excluded. It is necessary to point out that the systematic review conducted is of a qualitative nature; in other words, it is a review without meta-analysis and, consequently, the evidence has been presented in a descriptive manner and without statistical analysis. Moreover, the methodological quality of the studies/risk of bias was not evaluated. Finally, although the main objective of this systematic review was to analyse both the objective and subjective instruments used to assess the levels of PA in primary schoolchildren diagnosed with ASD, the possible effect associated to comorbities, which might influence the results of the tests, have not been analysed.

## 5. Conclusions

The accelerometer and the questionnaire were the instruments mainly chosen to measure the PA level (in terms of time and/or intensity) in children with ASD. Different models have been used in both instruments, but all of them, besides showing very similar results in terms of PA practice, have detected the same or similar drawbacks. In the case of the accelerometer, they highlighted the inability of the instrument to evaluate water activities and its difficulty to discern the functional movements from the stereotypical ones characteristic of ASD. The questionnaires, on the other hand, were qualified as useful instruments, but they were conditioned by the participants’ personal interpretation. As for the questionnaires, validity and reliability studies of these instruments should be carried out in populations with ASD.

The low levels of PA in the ASD group indicate the need for further research in this field, as well as the need to encourage PA practice through the promotion of sports programmes designed to meet the needs of children with the disorder. Especially the school environment, as it is one of the places where young people spend more time, should guarantee, by introducing the appropriate measures and adaptations, that all students understand PA as a means to favour personal and social development.

## Figures and Tables

**Table 1:** Search strategy in PsycINFO.

Category	Keywords
Autism Spectrum Disorder	Ti (ASD OR autism OR autism spectrum disorder) OR Ab (ASD OR autism OR autism spectrum disorder)
Physical Activity	Ti (physical activity OR physical exercise OR physical fitness OR physical condition) OR Ab (physical activity OR physical exercise OR physical fitness OR physical condition)
Measurement	Ti (assess* OR evaluat* OR measure* OR test* OR instrument assess*) OR Ab (assess* OR evaluat* OR measure* OR test* OR instrument assess*)
Population	Ti (primary educat* OR primary school OR elementar* school OR child*) OR Ab (primary educat* OR primary school OR elementar* school OR child*)

Ti: Title; Ab: Abstract.

**Table 2:** Description of the participants and methodology of the studies.

First Author and Year	Country	Participants		Design, Intervention	Objective
		Sample	ASD Diagnosis		
Pan, 2008 [20]	Taiwan	48 schoolchildren: ASD (23 boys and 1 girl) and Non-ASD (23 boys and 1 girl) aged 7–12 years old (14 schools).	Autism (mild or high functioning, *n* = 12; moderate, *n* = 9) and Asperger’s syndrome (*n* = 3). All without ID or severe behavioural problems. Manifest comorbidities: yes.	Follow-up during the regular school day (5 consecutive days). Periods of interest: school breaks (morning and afternoon). No specific intervention space was needed; the study was carried out in the participants’ environment.	To compare the percentage of time spent by children with and without ASD in moderate-to-high intensity PA during their inclusive school break time.
Memari et al., 2012 [21]	Tehran (Iran)	80 schoolchildren with ASD (52 boys and 28 girls) aged 7 to 14 years old (special schools). Exclusion of 10 students (malfunctioning device, *n* = 4; invalid data, *n* = 6).	High-functioning autism (CI > 70) without concomitant ID. Exclusion criteria: orthopaedic dysfunctions, inability to use/accept accelerometers and serious behavioural problems. Manifest comorbidities: yes.	Follow-up during 1 full week (7 days and 7 nights). Two comparisons of special interest: weekend; school day–extracurricular activity hours.	To analyse the patterns of PA in students with ASD, as well as their correlation with factors which may influence their activity levels.
Hinckson et al., 2013 [22]	New Zealand	17 schoolchildren: ASD (6 boys and 1 girl) and ID (4 boys and 6 girls) between 7 and 20 years old (2 special needs centres). All were obese or overweight.	Formal diagnosis of ASD. The degree of severity was not specified. Manifest comorbidities: yes.	School programme of 10 weeks dedicated to PA, nutrition and motivational skills. Data collection before and after the programme and 24 weeks after the interventionPresence and participation of families.	To determine the effectiveness of a PA and nutrition-based programme for weight control in obese schoolchildren with ID or autism.
Tyler et al., 2014 [23]	USA	29 schoolchildren: ASD (9 boys and 8 girls) and Non-ASD (6 boys and 6 girls) between 9 and 17 years old.	Formal diagnosis of ASD. The degree of severity was not specified. Manifest comorbidities: no.	Laboratory environment (diagnostic assessment, anthropometric measurements and assessment of physical fitness) and natural environment of students to measure PA (7 days).	To analyse the physical fitness and PA of schoolchildren with ASD, in comparison with their normotypically developing peers.
Memari et al., 2015 [24]	Tehran (Iran)	83 schoolchildren with ASD (52 boys and 31 girls) between 6 and 15 years old (4 specific schools for autism).	High-functioning autism (IQ > 70). Manifest comorbidities: no.	Family-based intervention aimed at finding out levels of PA practice in leisure time (7 days).	To assess the participation of children with ASD in daily physical activities, and their relationship to a range of factors.
Must et al., 2015 [25]	USA	111 schoolchildren: ASD (53, 83% children) and Non-ASD (58, 78% children) between 3 and 11 years old.	Formal diagnosis of ASD. The degree of severity was not specified. Manifest comorbidities: no.	Cross-sectional study carried out between January 2007 and December 2008.	To compare the prevalence of barriers towards PA among schoolchildren with and without ASD, as well as to assess the association between barriers, PA practice and screen time in children with ASD.
Haegele et al., 2018 [26]	USA	12 schoolchildren: ASD + VI (4 boys and 2 girls) and VI (4 boys and 2 girls) between 8 and 16 years old (school for visually impaired children).	Formal diagnosis of ASD. The degree of severity was not specified. The lack of accuracy when establishing the diagnosis may have prevented learning about additional limitations. Manifest comorbidities: no.	Intervention lasting 1 school week (5 days). PA level was measured during 4 days (Monday to Thursday, 8 h minimum). A physical fitness test was conducted on Friday during the physical education session.	To compare PA and physical fitness among schoolchildren with ASD+VI and those with only VI.
Bricout et al., 2018 [27]	France	40 schoolchildren (all children): ASD (20) and Non-ASD (20) between 8 and 13 years old.	High-functioning autism (CI > 70) without concomitant ID. Exclusion criteria: Contraindication for physical exercise, psychiatric or medical disorders, respiratory disorders and medical treatment. Manifest comorbidities: no.	Laboratory environment (respiratory capacity and physical fitness) and natural environment of students in order to measure PA (7 days).	To examine the cardiorespiratory fitness of children with ASD and compare it with that of normotypically developing schoolchildren.
Healy et al., 2018 [28]	Ireland	110 schoolchildren: ASD (55) and Non-ASD (55) aged 9. Girls represented 15% (*n* = 8) of both groups.	Formal diagnosis of ASD. The degree of severity was not specified.Manifest comorbidities: no.	Data from a nationally representative cohort study.	To compare the psychosocial factors associated with ASD and screen time between children with and without ASD.
Woodman et al., 2018 [29]	USA	13 schoolchildren with ASD (11 boys and 2 girls) between 5 and 13 years old (public school for students with ASD).	Formal diagnosis of ASD, with concomitant diagnosis of ID. Manifest comorbidities: no.	Jogging programme during 6 consecutive school days. The intensity of PA was analysed in a structured (from 9:50 to 10:25) and unstructured (from 10:25 to 10:45) jogging period, subject to different musical conditions.	To examine the influence of music on exercise intensity in children with ASD by means of a jogging programme.
Garcia et al., 2019 [30]	USA	14 schoolchildren with ASD (12 boys and 2 girls) aged 8 to 17 years old. Exclusion criteria: serious physical limitations and violent behaviour.	Formal diagnosis of ASD. The degree of severity was not specified. Manifest comorbidities: yes.	8-week Judo Programme to promote PA (summer; 1 session of 45 min per week).	To examine the effectiveness of a judo programme aimed at promoting moderate-to-high intensity PA and reducing sedentary behaviours in children with ASD.
Garcia et al., 2019 [31]	USA	49 ASD schoolchildren (36 boys and 13 girls) aged 8 to 17 (public school for ASD students). Exclusion of 1 student (non-compliance with accelerometer time criteria).	High-functioning autism (IQ > 70). Manifest comorbidities: no.	Follow-up during 1 week (7 days and 7 nights) to analyse the quality and duration of sleep and regular levels of PA.	To compare demographic and lifestyle factors with duration and quality of sleep in children with ASD.

**Table 3:** Measurement instruments of the studies.

First Author and Year	Measurement Instruments				
	Main Instruments		Complementary Instruments	Other Instruments	Description
	Objective	Subjective			
Pan, 2008 [20]	x	-	-	-	Objective instrument: GT1M ActiGraph (uniaxial accelerometer) programmed to collect data at 1-min intervals; tied on the right side of the hip (using an elastic belt). Its use was discontinued at the end of the school day; the data were downloaded immediately and it was restarted the following day.
Memari et al., 2012 [21]	x	-	x	-	Objective instrument: GT3X ActiGraph (triaxial accelerometer) programmed to collect data at 1-min intervals; tied on the right side of the hip (using an elastic belt). Complementary instruments: A logbook/log sheet (families and teachers) to record the hours when the device was not used. Questionnaire (families) to measure sedentary behaviour.
Hinckson et al., 2013 [22]	-	x	-	x	Subjective instrument: PA questionnaire (MEND) completed by the families. Selection of 2 out of the 4 questions on the MEND: time dedicated to activity/inactivity and time dedicated to moderate-to-high intensity PA. Other instruments: Nutrition questionnaire (14 items) on the frequency of having breakfast, carbonated drinks, white bread, wholemeal cereals, pastries and fresh food. 6-min walk test (6MWT) to assess physical fitness.
Tyler et al., 2014 [23]	x	-	x	x	Objective instrument: GTX3 + ActiGraph (triaxial accelerometer, valid for children with ID) tied on the right side of the hip (using an elastic belt). It was used at all times (except under the water and at night). The data collected were reduced to 4 categories (sedentary, low, moderate and moderate-to-high intensity). Complementary instruments: A logbook/log sheet (families) to record the hours when the device was not used. Other instruments: Fitness tests on aerobic capacity, muscle strength and flexibility.
Memari et al., 2015 [24]	-	x	x	-	Subjective instrument: PA questionnaire (adapted from GLTEQ) completed by families and teachers Two key topics: intensity and frequency of PA. Complementary instruments: List of barriers (families) against the practice of PA in leisure time. Daily activity journal (families) to know the type of game (individual or social).
Must et al., 2015 [25]	-	x	x	-	Subjective instrument: PA questionnaire (CHAMPS) completed by families in order to determine participation in structured and unstructured physical activities (type of activity and frequency). Complementary instruments: Screen time questionnaire (families) to determine the number of hours schoolchildren spent sitting or lying down (TV, video games, computer). Questionnaire on barriers (families) against PA practice (of the person, the family, social, community).
Haegele et al., 2018 [26]	x	-	-	x	Objective instrument: GT3X ActiGraph (triaxial accelerometer) that counts the accelerations at a sampling frequency of 30 HZ, tied on the right side of the hip (using an elastic belt). It was used only during the waking state hours and the data were classified into 4 categories (sedentary, low, moderate and high intensity). Other instruments: Fitness test (based on the Brockport test) to measure aerobic resistance, upper body muscle strength, abdominal muscle strength and flexibility.
Bricout et al., 2018 [27]	x	x	x	x	Objective instrument: Pro-3 SenseWear (triaxial accelerometer) placed on the triceps with an elastic belt to measure PA. It was used 24 h a day (except when showering or swimming). Subjective instrument: PA Questionnaire (PAQ-C) completed by families and schoolchildren (9 items). PA periods: week–weekend; with or without a teacher. Complementary instruments: A logbook/log sheet (families) to record the hours when the device was not used. Other instruments: EUROFIT battery to measure physical fitness.
Healy et al., 2018 [28]	-	x	x	-	Subjective instrument: PA questionnaire (GLTEQ) completed by families (based on the last 14 days; frequency, moderate-to-high or low intensity) and by students (based on the last 7 days; PA practice). Complementary instruments: Screen time questionnaire (families) with 3 variables (TV, computers and video games).
Woodman et al., 2018 [29]	x	-	-	-	Objective instrument: Omron activity device (Model HJA-750C; triaxial accelerometer) to measure METs (metabolic equivalent of task) and the percentage of 10-s intervals in high-intensity activity. It was placed on the back of the elastic waistband of school uniforms (so that when swinging the arms did not touch the accelerometer). It was used for 6 days (from 09:15 to 10:45).
Garcia et al., 2019 [30]	x	-	-	-	Objective instrument: GT9X ActiGraph (triaxial accelerometer which filters out high-frequency vibrations in order to artificially increase the data) programmed to collect data at 1-min intervals; placed on the non-dominant wrist (7 days). Data collected on the initial day were excluded from the analysis (possible artificial increase in PA).
Garcia et al., 2019 [31]	x	-	x	-	Objective instrument: GT9X ActiGraph (triaxial accelerometer) placed on the non-dominant wrist. It was used on a 24-h basis (except during water activities) to measure the quality and duration of sleep and the levels of PA. Complementary instruments: Screen time questionnaire (families) to determine the number of hours schoolchildren spent in sedentary activities (TV, computers, video games; 7-point scale).

**Table 4:** Main results of the studies.

First Author and Year	Results	Limitations	Conclusions
Pan, 2008 [20]	PA is stable, no group was moderately active for more than 50% of the break time. Schoolchildren without ID spent more time in moderate-to-high intensity PA when compared to students with ASD (36.15% vs. 27.2%). Subgroup ASD/Early primary (1st, 2nd and 3rd) more active than the group ASD/Late primary (4th, 5th and 6th).	Cross-sectional design of the studio. Reduced sample size. Presence of the accelerometer (possible rejection by the students). Data collection in 1-min intervals (possible underestimation of PA in free play). Absence of social, behavioural, cognitive and motor skills assessment (possible biases in the findings).	The lower level of PA in schoolchildren with ASD during breaks suggests that unstructured time during the school day may need to be redesigned for structuring. In order to improve the PA of students with ASD during the breaks, several types of strategies or interventions should be developed.
Memari et al., 2012 [21]	Significant differences between age groups and sex for PA (minimum level of PA among 13–14-year-olds and girls). More PA in out-of-school hours than during school No significant changes were found in PA levels comparing weekdays and weekends.	Cross-sectional design of the studio. Absence of a control group with which to compare the results. Homogeneity of the sample (difficulty to generalise the results to the ASD variety). Possible biases in the accelerometer results due to their incompatibility with aquatic activities and to the presence of stereotyped behaviours in schoolchildren with ASD.	The lower level of PA in schoolchildren with ASD during the school day underlines the need to revise school programmes for adapted PA. For a higher level of PA in children with ASD, they should be given PA opportunities according to their socio-demographic profile.
Hinckson et al., 2013 [22]	Unclear data, with trivial effects. Children more active after the implementation of the programme.	Reduced sample size. No control group with which to compare the results. Non-validated PA questionnaire for schoolchildren with ID. Winter and summer assessments (possible influence of the climate on PA levels). Absence of quantitative measurements.	A programme specifically designed for the needs of schoolchildren with ID and their families is needed. The programme should focus on healthy living rather than on managing overweight. The school may be the appropriate place to deliver such a programme. Appropriate tools need to be developed to accurately determine PA in young people with ID.
Tyler et al., 2014 [23]	Children with ASD are less physically active than their normotypically developing peers (less time in moderate-to-high intensity PA and higher rates of sedentary behaviour).	Uneven sample sizes and unmatched controls. Reduced sample size. Introduction of individual adaptations in the evaluations.	Children with ASD face well-known health disparities, so efforts to promote PA in schools, and through public health initiatives, must include this group.
Memari et al., 2015 [24]	Only 10 (12%) schoolchildren were physically active. Only 6% “often” participated in PA, whereas the majority, 85.5%, opted for the “never/rarely” response. Boys were more active than girls and the older they were, the more inactive they became. Preference for playing alone.	Cross-sectional design of the studio. Absence of a control group with which to compare the results. Measures based only on information from families or teachers (possible biases due to subjectivity).	Only a small percentage of children with ASD are physically active, and the financial concerns, lack of opportunities and socio-demographic factors are major constraints on their ASD.
Must et al., 2015 [25]	More barriers against PA in schoolchildren with ASD. The total number of barriers was inversely correlated with the number of hours and types of PA, but was directly related to total screen time.	Homogeneous sample (predominantly white and well educated). Exclusive use of qualitative assessment tests (possible subjectivity bias).	PA programmes designed to meet the special requirements of the ASD population and conducted by adults with specialised training are urgently needed. Systematic assessment of such programmes will contribute to the development of best practices regarding PA programming for children with ASD.
Haegele et al., 2018 [26]	Sedentary activities are predominant during the week. Non-compliance with the recommendation of 60 daily minutes of moderate-high intensity PA. The ASD/VI group was significantly less active than the VI group.	Reduced sample size. The severity of ASD and associated comorbidities were not recorded, so the presence of additional limitations which could influence PA is unknown. Biases may appear in accelerometer results as they do not record upper body PA. Intervention in a school with many opportunities for PA practice (possible overestimation of results and difficulty to generalise findings).	This study identifies a substantial need for health promotion among young people with ASD and visual impairment, and the results show the need to encourage intervention programmes to help improve these health-related variables.
Bricout et al., 2018 [27]	The control group was more physically active than the ASD group (not significant results in any of the tests: accelerometer and questionnaire).	Reduced sample size. Very specific ASD group (CI > 70) and absence of girls in the study (difficulty to generalise results).	The results of the study suggest that children with ASD are in worse physical condition than their peers without ASD. Furthermore, since poor physical condition is a reliable indicator of poor health outcomes, this study provides an important argument for the systematic implementation of PA programmes aimed at young people with ASD.
Healy et al., 2018 [28]	The ASD group is less physically active and less involved in sports than the control group. Boys (both groups) are significantly more active and more involved in sports.	The severity of ASD was not recorded (difficulty to generalise results). Exclusive use of qualitative measures (possible biases due to subjectivity).	The association between negative social interactions and lower levels of PA in children with ASD should be examined as well as the social interventions developed among schoolchildren with ASD and their peers in PA settings.
Woodman et al., 2018 [29]	Average time spent on intense PA of 16 min/day. More PA levels in the structured period. In general, slower music produced greater PA benefits. The exception was found in schoolchildren with fewer maladaptive behaviours and mild or moderate symptoms of autism, who were more motivated by fast music.	Reduced sample size. Homogeneity of the sample (difficulty to generalise the results to the ASD variety).Unusual daily exercise in the school (possible overestimation of results). Schoolchildren did not participate in the selection of songs (children with ASD may be particularly sensitive to music).	Based on the results of the study, music should be incorporated into physical education programmes for schoolchildren with ASD, as it can motivate this group to participate in PA in order to prevent obesity and other health problems. Additionally, jogging is a low-cost, low-risk intervention which can be easily implemented in an educational setting.
Garcia et al., 2019 [30]	The daily time spent in moderate-to-high intensity PA increased significantly after the judo session. After the programme, the number of participants who complied with the recommendations of 60 min of daily PA was increased from 4 to 8 and sedentary behaviours were reduced.	Reduced sample size. Short duration of the intervention (8 weeks). Absence of a control condition (Judo condition was not compared to an inactive condition or to a general exercise class).	The study proved the preliminary effectiveness of the judo programme to promote PA among young people with ASD, finding that 50% of the sample continued to participate in judo or a similar martial arts training after the programme. Further studies are needed to support these findings, but judo programmes could be well received by this group.
Garcia et al., 2019 [31]	Children who met the criteria of duration and quality of sleep had more minutes of moderate-to-high intensity PA and less time in sedentary activities.Only 15% of the sample met the sleep criteria.	Cross-sectional design of the studio. Very specific group of ASD (IQ > 70) (difficulty to generalise results). The stereotyped behaviours of schoolchildren with ASD could artificially increase the data collected by the accelerometer.	The results of the study showed the association of healthy lifestyle factors (HF) with longer sleep among young people with ASD. Participants’ compliance with the accelerometer protocol was high (only one participant did not meet the criteria for minimum time of use), suggesting that this method of objectively assessing ASD may be feasible in the ASD population.

## Data Availability

Data is contained within article.
